# Doing philosophy and the future of the ‘good doctor’ paradigm

**DOI:** 10.1007/s11019-025-10294-3

**Published:** 2025-08-19

**Authors:** Faye Tucker

**Affiliations:** Lancaster Medical School, Faculty of Health and Medicine, Health Innovation One, Sir John Fisher Drive, Lancaster University, Lancaster, LA1 4AT UK

**Keywords:** Medical education, Philosophical pedagogy, Artificial intelligence, Doctor-patient relationship

## Abstract

The author argues for the substantive *doing* of philosophy (as opposed to learning about it) as part of medical training. The paper presents a view of medical education as diminishing the critical thinking skills and humanistic values of future clinicians in favour of fact-recall and pattern recognition. This is due to increasingly assessment-driven curriculums and the need to meet extremely high, and rigorous, institutional and industry/sector standards. The author argues that current medical training favours a particular kind of learning, and therefore produces a particular kind of clinician, that may meet these standards and thrive in competitive and high-pressure practice but may not be best for patients. Furthermore, as artificial intelligence (AI) and emerging technologies rapidly change the landscape of medicine, current medical training may also not be best for these clinicians. The ‘good doctors’ that we are currently training, face a ‘survival of the fittest situation’ whereby they are no longer able to survive in a changing landscape, and therefore medical education is failing our future ‘good doctors’. Changes to the content and delivery of medical education need to happen now to mitigate this failing and give doctors: first, what they need to survive; and second, what they need to properly care for patients in a changing industry, increasingly served by AI. Doing philosophy has the potential to cultivate the thinking skills, inter-personal skills, personal attributes, and humanistic values needed by the ‘good doctor’ of the future.

I argue that the substantive doing of philosophy should be part of medical training. Making the case for a philosophical education is not new, and here I draw on these arguments to defend philosophy against increasing marginalisation by pedagogies and subjects that fit assessment-driven clinical training, and the reduction of the subject, where it is present, to ‘learning about’ as opposed to *doing.* The reason we should worry about this is that health care services are at risk of finding themselves in a ‘survival of the fittest’ situation where our ‘good doctors’ meet institutional and industrial expectations but are no longer necessarily best for *patients*. Furthermore, as emerging technologies, including artificial intelligence, are integrated into medicine, the environment in which doctors must practice will change significantly. Our thinking about what makes a ‘good doctor’ will need to adapt, and so must the medical curriculum, to best serve our future doctors.

## The ‘good doctor’ paradigm

Institutional expectations of the ‘good doctor’ are high. What is required of recruits, graduates and the workforce is, at best, optimistic and, at worst, unachievable. Furthermore, patient priorities for their interactions with clinicians do not necessarily match the institutional view. In this section, I give a sense of these two competing perspectives and conclude that this leads to two disparate ideas about what constitutes a ‘good doctor’.

In the UK, the Medical Schools Council (MSC) asks applicants to medicine to demonstrate a range of pre-existing skills, values and attributes, including academic excellence, resilience, empathy, conscientiousness, organisation, and communication skills (Medical Schools Council Selection Alliance 2018). These are assessed during a rigorous application process that screens academic high achievers at the interview stage. Applicants must demonstrate knowledge of the sector, an appreciation of what is entailed in a medical career and be decent human beings.

The General Medical Council (GMC) monitors students’ behaviour both in and out of the university and clinical setting, and students are subject to ‘fitness to practice’. Their probity is scrutinised throughout training, and they must demonstrate a commitment to their profession at all times. The GMC requires that graduates meet outcome expectations that range from diagnosis and medical management, to clinical research and leadership skills (General Medical Council 2020). In the UK, clinicians train for a minimum of 10 years, requiring enormous resilience and determination, the ability to learn and develop in high-pressure, competitive environments, and a willingness to accept compromise.

Once in practice, staff must be mindful of the National Health Service (NHS) core values, which form part of the NHS constitution. These capture expectations that clinicians must ‘cherish excellence and professionalism’, ‘respond with humanity and kindness to each person’s pain, distress, anxiety or need’, and must strive to get ‘the basics of quality of care—safety, effectiveness and patient experience—right every time’ (UK Department of Health and Social Care 2012). It is fair to say that institutional and sector/industry expectations are high.

We might, however, think that patients are the best judges of what makes a good doctor. One director of the Patients Association lists 5 things patients want from their doctors (Stone 2003). These are eye contact, partnership, communication, time, and appointments. Interestingly, diagnostic excellence, safety, or leadership do not feature in this particular list, and instead the emphasis is on humanistic values and interpersonal skills. What patients appear to want is a sense of being valued by their doctor. Patients’ ideas about good doctoring are somewhat at odds with the expectations of the MSC, GMC and NHS. It is therefore possible to be a good doctor by meeting a variety of expectations, depending on which view you take. Given this, I sketch two different views of what a ‘good doctor’ is:

Good doctor one: This doctor may not always get to the bottom of patient problems, with a diagnostic success rate of somewhere in the region of 65%. Despite this, they always listen to their patients, genuinely care, and relate with empathy. Patients might say, **‘I’ve been seeing them for a while now and we’re still not sure what the problem is, but they take such an interest in what matters to me and listen to my worries. They are such a good doctor.’**

Good doctor two: This doctor has a high diagnostic success rate. Their manner is efficient and, though some patients may think them rude, their clinical excellence means they are very successful, and patients leave pleased with outcomes. Patients might say, **‘They don’t have time to chat, but I don’t take it personally, that’s their manner with everyone. My condition has massively improved. They are such a good doctor.’**

These ‘good doctor’ descriptions are central to my argument for the substantive *doing* of philosophy as part of medical education. In the next section I make clear what I mean by this.

## A distinction: learning about versus doing

Imagine trying to learn woodworking by reading the *Encyclopaedia of Woodwork* and never picking up a chisel. This simple analogy encapsulates an important distinction; there is a difference between ‘learning about’ and ‘(learning by) doing’. When you have read the entire *Encyclopaedia of Woodwork*, you have found out about the history of woodworking, the techniques that artisans use, and the creative pieces they have crafted—you have learned about woodworking. However, if you have never picked up a chisel, it is highly unlikely you will be proficient at woodworking by *learning about* it. It is only by working with wood that you will develop proficiency in the craft.

By analogy, when you have found out about the history of philosophy, the arguments that philosophers have made, and the conclusions they have drawn, you have learned about philosophy. *Doing* philosophy, on the other hand, is a practical activity. In fact, we could liken philosophy to a craft; it is a process of developing practice, honing skills, and something that, like a craft, is valuable for self-actualisation (Clark 2017).By *doing* philosophy, I intend the activity of philosophising. What philosophy is, and how it is done, is itself a question discussed by philosophers, appreciating the breadth and complexity of the philosophical project, and the many approaches and traditions within it (I am an analytic philosopher, that sits within a Western tradition of philosophy). It is not entirely stretching the craft analogy to claim that in woodworking and philosophy there is huge variety within both activities, but nevertheless family resemblances that help us classify them.

For the purposes of this paper, I settle on describing the activity of philosophy as: seeking to understand the world and human experience by a particular method or set of methods, that should include critical analysis of ideas (for example intuitions, problems, or concepts). Analytic philosophy unpacks what we think we know into its constituent parts, explains what these are and how they relate, and then rebuilds and tests new hypotheses through, for example, logical argument, counterexample, or thought experiment, with the aim of refining some truth/s. This is just one view of what doing philosophy is, which I hope is broad enough to capture some of what most philosophers are doing.

Much as the term ‘science’ captures both a method and a body of knowledge, the term ‘philosophy’ captures what it is to engage in a type of enquiry characterised by a philosophical method, as well as a body of knowledge. ‘Philosophy’ is an umbrella under which we find a wide range of sub-disciplines including metaphysics, epistemology, philosophy of science, political philosophy, and philosophy of medicine, all of which would have something valuable to contribute to a medical degree programme, if time and space were not at a premium. Ethics is another branch of philosophy, and, like other areas of philosophy, ethics is something we do as well as a body of knowledge. On most medical programmes, knowledge of ethics is thought to be important learning, and indeed knowing the frameworks that shape the ethical, legal, and professional expectations of good medical practice is certainly core to clinical training. Beyond these ‘basics’ Zbigniew Zalewski argues that teaching philosophy to medical students is ‘of the upmost importance’ and that ‘It should fulfil at least three essential conditions: ‘(1) introduce future doctors and other medical professionals into the world of philosophical problems and philosophical thinking in order to broaden their mental horizons; (2) induce or evoke critical thinking as to undermine this approach to medicine which assumes biomedical knowledge to be a kind of secular revelation; (3) make thereby so educated people sensitive to patients as vulnerable persons and not only vulnerable bodies’ (Zalewski 2000). Zalewski is concerned that the biomedical model of educating medical students deliberately rejects the first two conditions and ignores the third, therefore leaving a significant gap in medical training. Additionally, Zalewski argues that familiarity with the complexities of philosophical thinking enables students to better comprehend and integrate the other humanities and social sciences into their biomedical knowledge, which would indicate philosophy can provide benefits to students’ studies beyond simply learning about ethics. However, the core teaching and assessment priorities on many medical programmes are the medical sciences and clinical skills necessary for students going into practice. There is reliance on the ‘hidden curriculum’ to inculcate values, inter-personal skills, and professionalism. Where philosophy and ethics do feature in medical training, it is often limited in scope and content. The focus is on the learning of principles to structure ethical decision-making (which somewhat miss the point of how we actually *make* ethical decisions), basic engagement with, and application of, key concepts in medicine (without valuable conceptual analysis), and a nod towards the intersection of ethics with medical law (resulting in limited abilities in *interpreting* the word of the law).

Furthermore, there are few institutional guidelines about how doing *philosophy*, as distinct from learning about *ethics*, should feature in the medical curriculum, nor is philosophy a universal offer across all institutions. The Institute of Medical Ethics (IME) provides guidance on what is necessary to prepare medical students for becoming a Foundation Doctor (IME 2019). The focus is on ‘ethics’ but many of the learning objectives ask students to be able to ‘evaluate’, ‘reflect on’, ‘consider’, ‘assess’, ‘analyse’, and ‘critically examine’, suggesting that the emphasis should be on *doing* rather than *learning about* ethics. In fact, the IME advocates for opportunities for students to discuss and explore concepts, and practice their ethical decision-making in risk-free settings which, in theory, could develop their philosophical skills. The suggestion is that small group teaching and cases are used as a starting point for this exploration, but there is no guidance on how to spot good philosophy in action, or how best to train and develop philosophical thinking.

The picture I have painted might sound fairly bleak, but these could be happy days for philosophy in medical education. The academic medical curriculum is dense, assessment driven, and runs alongside intense and high-pressure vocational training. As a result, the emphasis is on memorising, fact-recall, and pattern-recognition, and not on *thinking* (Lipman 1976). Non-medical science subjects, which present students with the best opportunity for critically and creatively engaging with material at conceptual depth, are already squeezed within the curriculum, but are at risk of being strangled altogether (Halperin 2010). Last year saw the introduction of the Medical Licensing Assessment (MLA) for graduate trainees where a minority of questions will test these aspects of the curriculum (General Medical Council 2024). This presents an obvious case for *reducing* the weighting of non-medical science subjects in the timetable. An added concern is that the MLA question format is ‘single best answer’ (a version of the multiple-choice question) which does not lend itself to testing the *understanding and application* of the concepts and principles that we teach our students. In an educational environment where learning is already instrumentalised, there is good cause to be concerned about the future of thinking skills in medical education, and it would be unsurprising to see further dumbing down, and reductionist approaches to learning about philosophical and ethical topics that fit with assessment methods.

## The case for *doing* philosophy

But why should we care? Exactly what is the point of doing philosophy as part of medical training. As noted, at least some institutions manage to produce doctors that presumably meet the high institutional professional standards with no philosophical training. On the other hand, others have recognised that taking an alternative pedagogical approach develops empathy, compassion, and open-mindedness, all attributes required to properly engage with patients as people and deliver authentic care. These attributes align with GMC expectations for graduates which identify a range of affective and social skills required for effective team working and for building appropriate relationships with patients to deliver quality care (GMC 2020).

The central claim in Rita Charon’s work on narrative medicine is that exploring and understanding stories develops students’ capacity to engage with and reflect on patients’ experiences of illness and therefore be more deeply connected to patients (Charon 2001). Narrative medicine as a practice, does resemble the mechanics of narrative *ethics—*which engages with narrative to problematise and contextualise moral questions and decisions—and so shares features with a very particular type of philosophical method. Indeed, Charon’s work is widely accepted and has been embraced by medical educators. However, I am arguing for something different.

Drawing on arguments that defend taking a philosophical approach in education, I defend the practical activity of *doing* philosophy as part of medical *training.* I argue that doing philosophy collaboratively, with others, can do a lot of the work of developing the skills for relating to patients and the core skills needed for patient diagnosis and treatment: ethical reasoning (including an appreciation of others’ perspectives, non-judgment, and the ability to change one’s mind), decision-making (including critical thinking and problem-solving), and analytical skills (including conceptual and abstract thinking). Studies have shown that doing philosophy in educational settings, in the form of collaborative philosophical enquiry, can have a positive effect on creative thinking and reasoning skills, including scientific reasoning (Camhy and Iberer 1988; Sprod 1997). Other evidence shows a positive impact on social values and self-esteem (Gardner 1999) and on affective and social skills (Millett and Tapper 2012). In addition to being generally desirable, the diverse range of skills that are developed by doing philosophy align with, or support achievement of, the GMC Outcomes for Graduates across the three areas of ‘Professional Values and Behaviours,’ ‘Professional Skills’, and ‘Professional Knowledge’ (GMC 2020).

There is pedagogical value to doing philosophy because it develops fundamental dispositions and thinking skills. Indeed, as John Dewey argued, ‘philosophy may even be defined *as the general theory of education’* (Dewey 1944). The Socratic method, often likened to children’s questioning to understanding, can be seen throughout education, not least in seminar-style group learning in higher education. However, it is overshadowed by methods that ‘teach to the test’ and encourage memorising and fact-recall. The educational movement, Philosophy for Children (P4C), explicitly disrupts traditional educational settings so children can access all aspects of the curriculum through Socratic collaborative enquiry (Sharp 1987a, b). It focuses on developing thinking skills, which the Society for the Advancement of Philosophical Enquiry and Reflection in Education (SAPERE) categorise into the 4Cs; caring, collaborative, creative, and critical (Sapere 2022). Recent iterations of P4C have engaged the ‘community’ more widely, and it is now used in colleges and universities, prisons and even on ‘the street’ to interrogate learning, ideas, and experiences. Doing philosophy with others also develops reflexive skills such as self-confidence, self-worth, self-esteem, self-efficacy and self-knowledge (Sharp 1987b). These are fundamental for a person to make and articulate a decision, because to do so they must reflect on what matters to them, endorse or reject values and options, and be able to communicate their choice to others. The overemphasis on memorising and pattern recognition in medical education is fertile ground for philosophy to step in and develop the right kind dispositions and thinking skills in clinicians.

Furthermore, and building on the reflexive skills outlined above, doing philosophy develops a sense of personhood or self (Sharp 2014). Dialogue with others about things that matter, enriches our value landscape. Without a wide horizon of values, our ability to properly reflect on our own values is reduced (Anderson 1993). Consider, for example, the echo chamber of social media, reflecting back our pre-existing views, so validating what we thought we already ‘knew’ rather than offering a perspective from which to evaluate our views and either endorse or reject them. The capacity to change our minds about what we think is good or bad, or compare how we live our lives with other possibilities, is both central to selfhood and to becoming autonomous rational agents.

Finally, to consolidate these arguments, doing philosophy is a kind of training that fits with the vocational aspect of medical education. Matthew Lipman, drawing on the work of Aristotle, argues that doing philosophy develops *phronesis* (Lipman 1991)*.* The concept of *phronesis* is common in Ancient Greek philosophy, most prominently in the work of Aristotle (Aristotle and Crisp 2014). Often translated as ‘practical wisdom’ it is a type of wisdom that is needed to *know what to do in practice*. In the work of Aristotle, we see the cultivation of particular habits and traits alongside *phronesis,* so that virtuous action becomes something that virtuous people do because they are virtuous and just know what to do. People become virtuous through practice, emulating the virtue they see in the virtuous, until they have *phronesis* and then know how to be virtuous without having to look to others for guidance—a kind of ‘fake it ‘till you make it’ of moral agency. When a virtuous character is developed, navigation of the moral world, relationships, and decision-making, are achieved by the virtuous through *phronesis.* Aristotle’s account of virtue is *training* to become a moral agent who knows what to do, and we surely would value doctors who have developed *phronesis.*

I have outlined established arguments for doing philosophy that are based on the activity having intrinsic value. Incorporating philosophical pedagogy into the suite of pedagogical methods used to deliver the full medical curriculum, including the medical sciences, has the potential to support a wide range of interpersonal skills, develop empathy and a caring, respectful disposition, and give clinicians confidence in making and communicating difficult decisions.

But there is a risk that I find these arguments compelling because I am a philosopher—my argument that everyone should do philosophy (because I do philosophy and they should value what I do), could be philosophical hegemony. Indeed, I do think doing philosophy is intrinsically valuable! But we need a more compelling argument to convince students who are already at capacity, and understandably assessment-focussed, as well as a sector/industry that is under-resourced, under-staffed, and over-burdened. The skills, values and attributes developed by philosophical training cannot be easily measured or assessed, and even though they might be *desirable,* they might not be *necessary* for being a doctor in the current environment because, as we have seen, there are a range of different ways to be a ‘good doctor’ that emphasise different skills and dispositions*.* What I present next is an argument that shows why doing philosophy is *instrumentally* valuable, especially as the current environment in which doctors practice and patients find themselves is undergoing potentially seismic change. On the terms of being instrumentally valuable, a philosophical curriculum is commensurable with, and can compete with other, outcome focussed, curriculum models. I will show that doing philosophy, and all that comes with it, turns out to be *necessary* to prepare our future doctors.

## Survival of the fittest: the future of the ‘good doctor’ paradigm

In this section, I begin by imagining what might happen if we do away with philosophy all together, while assuming the environment of clinical practice will remain unchanged. I have called this a ‘survival of the fittest’ situation, akin to Charles Darwin’s theory of evolution, which describes that each species’ generation adapt to their environment, in particular to scarcity and stress, and therefore become better able to cope; those species that change in response to the world around them survive, those that do not become extinct.

First, a reminder of the two disparate ideas about ‘good doctor’, presented in section one:

Good doctor one: **‘I’ve been seeing them for a while now and we’re still not sure what the problem is, but they take such an interest in what matters to me and listen to my worries. They are such a good doctor.’**

Good doctor two: **‘They don’t have time to chat, but I don’t take it personally, that’s their manner with everyone. My condition has massively improved. They are such a good doctor.’**

What will happen to our good doctors if medical education becomes even more assessment-driven, and the clinical sector remains the ultra-high-pressure environment that it has become? First, let us assume that good doctor one, who is less diagnostically accurate, may not do as well in their exams. Their warmth and empathy cannot not easily be assessed and will not be cultivated or valued within a medical education that has squeezed out authentic opportunities for self-building and reflection, in favour of memorising lists of information, formulaic communication techniques, pattern-recognition, and technical excellence. Less doctors like good doctor one will get through training. Second, if good doctor one *does* get through their training, they will be working in an environment where there is limited time to see patients who may have been waiting days, weeks or months to see a doctor, as well as incredible pressure to correctly diagnose in a landscape of stretched resources and long waiting lists. Patients may prioritise good doctor one, but the sector/industry will not. Likely, it is these doctors, that see patients as people, want to take time to work with them, and have a caring disposition towards them, that feel stressed and pressured by the system and are most susceptible to burnout (Graves et. al. 2023). Not only will good doctor two do better in this environment, but good doctor one may leave the sector all together. The end of the story for good doctor one is extinction.

Adaption of the good doctor paradigm to the current educational and sector/industry climate means that our future ‘good doctors’ will be like good doctor two. This is obviously bad for good doctor one. This may also be bad for patients, given patient perspectives of a ‘good doctor’ presented in section one. Importantly however, the clinical environment *is* in fact rapidly changing. For this reason, the good doctor paradigm shift is also bad for the good doctor two, unless our medical education also changes to meets the needs of a changing environment, as I go on to explain.

## Future medicine, artificial intelligence and the good doctor two

AI is going to change the medical service industry, and soon. AI is already changing diagnostic tasks, and current recruits to medicine degrees are likely to work in a landscape that is unrecognisable from the one that is familiar to them now (Alrassi et al. 2021). AI will change how patients are triaged and communicated with, how samples are tested, and how diagnoses are made, but caring will be less easily replaced by technology (Johnston 2018). Future forecasts of the impact of emerging technologies, perhaps especially AI, on medical practice ‘highlight the importance of humanistic characteristics and behaviors—critical human skills—for practitioners’ (Alrassi et al. 2021). But there will be other impacts on the ethical dimensions clinical practice. Our future clinicians will need the skills and confidence to manage technology and data responsibly and will have to engage with new ethical and legal questions that may challenge our accepted principles (Arnold 2021), but will likely still be able to offer a uniquely human contribution to shared decision-making and patient experience, in other words ‘the nonanalytical, humanistic aspects of medicine—most importantly, the art of caring…’ (Johnston 2018), by advocating for underprivileged groups, and communicating the risks, as well as the benefits, of AI to their patients with honesty and compassion (Svensson and Jotterand 2022).

Further away in the future, AI may display (but perhaps not *experience*) humanistic values in the same way as good doctor one (Svensson and Jotterand 2022). However, what I am describing is the threat that AI poses to good doctor two, and it is real and approaching fast. Already AI is used to regurgitate lists of information, streamline effective communication where appropriate, recognise patterns, and provide technical excellence in some diagnostics—all the things that are favoured in current medical training and that Good Doctor two does so well. What we should anticipate is that AI will likely do this better, faster, more reliably and crucially, more *economically*, than good doctor two. As S. Claiborne Johnston writes, ‘[t]he medical students of today will practice in a world where information technology is sophisticated and omnipresent. In this world, the tasks of memorization and analysis will be less important to them as practicing physicians.’ (Johnston 2018). AI will potentially do better than humans at many of the analytic aspects of patient diagnosis and treatment. Our doctors must develop skills to thrive in the gaps that are left, and the skills to adapt to, and responsibly manage, systems that may be, as yet, unimaginable.

Currently, medicine is practiced in an environment that is evolving more quickly than our doctors can adapt and we are at risk of training a workforce of professionals ill-equipped to survive. Philosopher, Bertrand Russell wrote, ‘[t]o teach how to live without certainty, and yet without being paralysed by hesitation, is perhaps the chief thing that philosophy, in our age, can still do for those who study it’ (Russell 1996). By building philosophy into medical training, the aim is not to create a generation of philosopher-physicians, but to create thinkers. Specifically, the resilient clinician who will thrive in an uncertain future, will not only know *what* principles inform their practice, but will have the depth of understanding, and crucially the skills, to be able to *interrogate* and *apply* those principles in new contexts as they emerge, and engage in developing ethical approaches to resolving the unknown unknowns of the future (Niet and Bleakley 2021). Indeed, there is much uncertainty about what technology might emerge, and how this will change clinical practice, but also about how we should manage these changes responsibly, with fairness and beneficence. Integration of AI into clinical practice has been happening over several years in a regulation vacuum, and only now are more indicative guidelines available (WHO 2023, 2025), despite there being reasons to tightly regulate the development and use of AI systems in healthcare (Svensson and Jotterand 2022). As with many technologies, ethical guidelines for best responsible AI governance and use in health and medicine are not able to keep pace with the advancing technology, and practitioners may lack training and confidence. Over 93% of medical student participants in one study wanted training in solving ethical problems in AI (Civaner et al. 2022), which demonstrates a perceived need for preparation for ethical problem solving in unknown or uncertain situations, even though we might assume the medical students who took part in the survey would have some knowledge of the ethical principles that underpin clinical decision making. Using philosophical tools to reflect on, and engage with, challenges and resolve problems is seen by some ‘as the fundamental approach to pausing at times of complexity and uncertainty to ask basic questions about seemingly obvious practices so that we can see (and do) things in new ways' (Veen and Cianciolo 2020).

## Recommendations

Some have suggested that steps to ensure that doctors are prepared for an AI future should begin with the selection process for medical school (Alrassi et al. 2021), and there is scope to further hone screening processes to this end. However, the focus of this paper is on how training in medical education can further develop the skills and dispositions that will be needed in the AI-integrated clinical landscape of the near future. In summary, there have been calls for medical education to prepare students by: providing guidance on the knowledge and application of emerging ethical issues (Katznelson and Gerke 2021; Svensson and Jotterand 2022); developing humanistic skills (Han et al. 2019; Halperin 2010; Lee et al. 2021); enhancing team building, problem-solving, and shared decision-making (Johnston 2018; Han et al. 2019; Alrassi et al. 2021); and, giving them tools to manage uncertainty (Tonelli and Upshur 2019). I have argued that integrating philosophical pedagogy, in particular collaborative enquiry, into the delivery of the medical curriculum has clear benefits, is one way that medical education can meet these challenges, and prepare medical students for rapidly changing clinical practice. However, given the demands on the medical degree, both academic and vocational, any recommendations must be readily accommodated within an already crowded space. Indeed, an integrated approach would both be practical, but also pedagogically preferable, to fully embed philosophy into the medical curriculum.

In line with the arguments I have presented, Pekka Louhiala argues that a philosophical attitude is perhaps more important than philosophical theory (2003). Nevertheless, there is clearly a requirement for some learning and understanding of the principles that underpin ethical clinical practice. If philosophical enquiry is a craft, then logic and language are the tools of that craft, and so time should be given over to teaching a toolkit for critical thinking. This might include specialist teaching of argumentation, good and bad reasoning, and common informal fallacies. This should also include specialist teaching of core concepts, as a basis for exploration, interrogation, and application.

We must avoid telling students what to think and instead give them space to develop confidence in thinking for themselves in the face of new professional challenges. The adoption of a philosophical approach to learning across the curriculum, developing activities that critically engage with concepts and material and test out scope and meaning through application, as opposed to rote learning, memorising, and fact recall, will offer students opportunities to use their philosophical skills.

Ideally, this should happen in collaboration with others, and staff that facilitate collaborative enquiry would benefit from training in fostering collaborative thinking, respecting others’ views, disagreeing respectfully, and recognising good from bad arguments. Evidence suggests that training educators in collaborative philosophical enquiry can support them to facilitate students in a problem-based curriculum and enable connected learning between disciplines for learners (Scholl et al. 2015). With suitable training in philosophical pedagogy for educators, alongside specialist core teaching, students could be engaged in the philosophical questions that arise from their multi-disciplinary studies as part of knowledge exploration and application, and from their daily clinical practice, as part of reflection and self-building.

Building on philosophical foundations, complex simulated or scenario-based problem-solving in groups, debates, discussions, and meaningful team-based decision-making might be useful approaches. This pedagogical approach can be embedded as part of a suite of pedagogical approaches right across the medical curriculum, from medical humanities and the social sciences, through to medical science subjects, and especially within medical ethics. Instead of telling students what it means to treat someone with dignity, for example, we can design a series of learning opportunities that include space for students to share their own views, challenge conventional wisdom, and debate what it means to treat someone with dignity across a series of challenging or controversial contexts that might sit at the extremes of their conceptual understanding. This stretches depth of understanding and requires, beyond simple application of a principle, the testing of the very concept itself, while developing that practical wisdom, or *phronesis* that would seem to be a desirable characteristic of the resilient clinicians of the future. Collaborative philosophical reflection on clinical practice would engage peers in recognising when mistakes are made, challenging bias with confidence and humility, and presenting coherent reasons to justify positions. These are all powerful skills needed in clinical decision making, will be required to navigate responsible AI use, and develop the humanistic dispositions of empathy, compassion, perspective taking and respectful communication.

## Conclusion

In summary, this paper has argued that the substantive doing of philosophy should be part of medical training. While there is increasing focus on ‘teaching to the test’ in assessment-driven clinical training, and the reduction of philosophy to ‘learning about’ and not *doing,* medical educators are failing both patients and future doctors themselves. Changes to the content and delivery of medical education needs to happen now to mitigate this failing and give doctors: first, what they need to survive; and second, what they need to properly care for patients in a changing industry, increasingly served by AI. Here, I have made the case that doing philosophy has the potential to cultivate the thinking skills, inter-personal skills, personal attributes, and humanistic values needed by the ‘good doctor’ of the future.
